# Research on an Ankle Joint Auxiliary Rehabilitation Robot with a Rigid-Flexible Hybrid Drive Based on a 2-S′PS′ Mechanism

**DOI:** 10.1155/2019/7071064

**Published:** 2019-07-17

**Authors:** Caidong Wang, Liangwen Wang, Tuanhui Wang, Hongpeng Li, Wenliao Du, Fannian Meng, Weiwei Zhang

**Affiliations:** ^1^School of Mechanical and Electrical Engineering, Zhengzhou University of Light Industry, Henan Provincial Key Laboratory of Intelligent Manufacturing of Mechanical Equipment, Zhengzhou 450002, China; ^2^School of Logistics Engineering College, Shanghai Maritime University, Shanghai 200000, China

## Abstract

An ankle joint auxiliary rehabilitation robot has been developed, which consists of an upper platform, a lower platform, a dorsiflexion/plantar flexion drive system, a varus/valgus drive system, and some connecting parts. The upper platform connects to the lower platform through a ball pin pair and two driving branch chains based on the S′PS′ mechanism. Although the robot has two degrees of freedom (DOF), the upper platform can realize three kinds of motion. To achieve ankle joint auxiliary rehabilitation, the ankle joint of patients on the upper platform makes a bionic motion. The robot uses a centre ball pin pair as the main support to simulate the motion of the ankle joint; the upper platform and the centre ball pin pair construct a mirror image of a patient's foot and ankle joint, which satisfies the human body physiological characteristics; the driving systems adopt a rigid-flexible hybrid structure; and the dorsiflexion/plantar flexion motion and the varus/valgus motion are decoupled. These structural features can avoid secondary damage to the patient. The rehabilitation process is considered, and energy consumption of the robot is studied. An experimental prototype demonstrates that the robot can simulate the motion of the human foot.

## 1. Introduction

Many studies have shown that high-intensity repetitive movements play an important role in the effectiveness of robot-assisted therapy [1]. Some ankle rehabilitation robots for treating ankle injuries have been developed. For example, Roy et al. [2] developed a three-DOF wearable ankle robot, back-drivable with low intrinsic mechanical impedance actuated by two actuators. Saglia et al. [3] designed a redundantly actuated parallel mechanism for ankle rehabilitation. Yoon and Ryu [4] presented a reconfigurable ankle rehabilitation robot to cover various rehabilitation exercise modes. Jamwal et al. [5] designed a rehabilitation robot with three-DOF rotation. The robot has four actuators. Girone et al. [6] used a Stewart platform-based system as an ankle robot with six DOFs. Veneva [7] introduced an ankle-foot orthosis with one DOF for the foot segment and another one for the shank segment. Agrawal et al. [8] designed a two-DOF orthosis with pronation-supination and flexion-extension movements. Bi [9] proposed a spherical parallel kinematic machine as an ankle rehabilitation robot, which can improve the adaptability to meet the patient's needs during rehabilitation. Lu et al. [10] proposed a three-DOF ankle robot combining passive-active training. Aggogeri et al. [11] proposed a new device based on a single-DOF parallel mechanism able to perform trajectories similar to the patient's ankle. Erdogan et al. [12] presented a configurable, powered exoskeleton for ankle rehabilitation. Liao et al. [13] proposed a novel hybrid ankle rehabilitation robot, which is composed of a serial and a parallel part. The parallel part of the robot was simplified as a constrained 3-PSP parallel mechanism. The kinematic analyses showed that the proposed hybrid rehabilitation robot can not only realize three kinds of ankle rehabilitation motions but also eliminate singularity with enhanced workspace.

Nowadays, research on ankle joint robots involves several aspects, including control, torque, motion planning, and optimization. For example, Rosado et al. [14] implemented PID controllers in the development of passive rehabilitation exercises. Meng et al. [15] presented a robust iterative feedback tuning technique for repetitive training control of a compliant parallel ankle rehabilitation robot. Zhang et al. [16] proposed a computational ankle model for use in robot-assisted therapy estimating the passive ankle torque. Ayas et al. [17] designed a fractional-order controller for a developed 2-DOF parallel ankle rehabilitation robot subject to external disturbance to improve the trajectory tracking performance.

At present, a number of rehabilitation robots are under investigation. However, only very few rehabilitation robots have been commercialised [9, 18, 19]. Rehabilitation robotics is penetrating the market very slowly. The significant limitations are the high cost and the difficulty to meet some specific needs from patients. For low-income and middle-income classes, only 5-15% of people who need assistive devices and technologies have access to these technologies [20]. There is a shortage of personnel trained to manage the provision of such devices and technologies. However, the research and development on rehabilitation robots is emerging due to the fact that the cost of excluding people with disabilities from taking an active part in community life is high and the improvement has to be borne by society, particularly for those who take on the burden of care. The following conclusions have been drawn from the literature reviews [9]:

1. The existing rehabilitation robots have unacceptably high price. Even though limited rehabilitation robots are commercially available, most of them are still placed at research institutes due to the lack of market attraction
2. Most of the existing ankle rehabilitation robots have coupled motions other than ankle rehabilitation needs. On the one hand, it increases the development cost since unnecessary redundant motions are used. Most importantly, due to the coupled translations, additional support will be required to endure the patient's weight. For example, a few ankle rehabilitation robots have coupled motions of the legs. It becomes very inconvenient for a patient to sit down and concentrate on the ankle rehabilitation
3. Most of the existing rehabilitation robots are designed for hospital environments

There is no indication that patients can operate and tailor the rehabilitation routines to their own needs. A completely new control mode is in demand which will allow a patient to operate the device by themselves and in the home environment.

Therefore, the following three points have been considered in our designed rehabilitation robot to further improve their performance and reduce the manufacturing and use costs simultaneously:

1. Among the three allowed motions of the human ankle, only dorsiflexion/plantar flexion and varus/valgus are considered, as they are more important for ankle rehabilitation [21]. The basic idea is thus to develop a rehabilitation robot primarily intended for the above two motions to meet some special patients' need and further reduce the cost of production and use
2. The objective is to design a robot in which the motion is fully decoupled into motion segments, to avoid the associated motion of multidrive motors
3. For existing robots, the motion law of rehabilitation needs to be further researched. In fact, by applying inappropriate rules while rehabilitating the patient, the exercise would be less effective and may lead to secondary damage to the patient

Based on these considerations, a bionic ankle joint auxiliary rehabilitation robot based on a 2-S′PS′ mechanism was designed.

The main innovation points include

1. The robot is designed with a special structure configuration. Between the upper platform and the lower platform, the centre sphere-pin pair and the two drive branch chains used for support are designed into a right triangle. Among them, the centre sphere-pin pair is a right-angle vertex, and the two drive branch chains are the vertexes of the two right-angle edges of the right triangle. For each drive branch chain, the spherical pin shafts of the two sphere-pin pairs are arranged along the direction of the right-angle side of the triangle. The spherical pin shaft of the centre sphere-pin pair is also arranged along the direction of another right-angle side of the triangle. Using this innovative structure configuration, the upper platform realizes dorsiflexion/plantar flexion and varus/valgus motions through rotation around two right-angle sides of the triangle
2. To make the robot motion be completely decoupled during dorsiflexion/plantar flexion or varus/valgus motions, dorsiflexion/plantar flexion and varus/valgus driving systems of the robot adopt the rigid-flexible hybrid structure. The two drive branch chains have the same structure. Each branched chain consists of a motor, slider block, spring, and others. When Motor I rotates to change Branch Chain 1 which results in motion of the upper platform, Branch Chain 2 will change its length to fit the upper platform motion. The compression of springs on Branch Chain 2 is large enough to compensate for this kind of change; therefore, Motor II on Branch Chain 2 keeps stationary. The same circumstance occurs for Motor II rotating to change Branch Chain 2
3. The robot uses a centre ball pin pair as the main support to simulate the motion of the ankle joint; a structure consisting of the upper platform and the centre ball pin pair is a mirror image of a patient's foot and ankle joint, which satisfies the human body physiological characteristics
4. The speed, acceleration, and energy consumption of a typical rehabilitation exercise are considered to select different motion laws for the upper platform of the robot, for applying appropriate rules while rehabilitating the patient and avoiding secondary damage to the patient

Table 1 shows actuation, range of motion (RoM), and motion decoupled characteristics about our designed robot and some stationary ankle rehabilitation robots. From Table 1, only our robot is completely decoupled in dorsiflexion/plantar flexion or varus/valgus motions.

The rest of the paper is organized as follows: In Section 2, the structure of the robot is presented. In Section 3, the kinematic model is established, and the workspace is calculated in Section 4. The motions of the robot's upper platform are simulated and analyzed under different motion laws in Section 5. The control system and the experimental research are discussed in Section 6. Conclusions are outlined in Section 7.

## 2. Structure and Working Principle of the Robot

According to the anatomical structure of the human ankle, the ankle involves a total of three kinds of motions, i.e., dorsiflexion/plantar flexion, varus/valgus, and adduction/abduction. Among them, dorsiflexion/plantar flexion and varus/valgus are the two most important [21].

**Figure 1:**
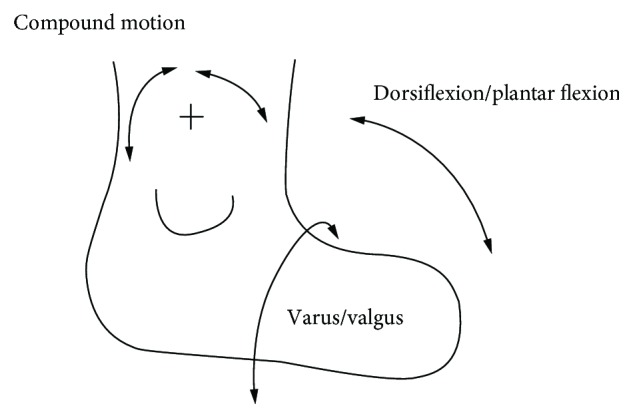
Schematic of the human ankle joint.

Therefore, an ankle joint auxiliary rehabilitation robot is designed according to the schematic shown in Figure 1. The robot with two drives and two DOFs is capable of three kinds of motions, namely, the dorsiflexion/plantar flexion motion, varus/valgus motion, and compound motion. The robot can be used by patients to exercise all these three motions. For patients with foot droop and lower limb muscle atrophy, the rehabilitation train can recover the ankle activity to normal, improve the muscle strength of lower limb muscle atrophy, and make the patient stand and walk during the rehabilitation period [28].

**Figure 2:**
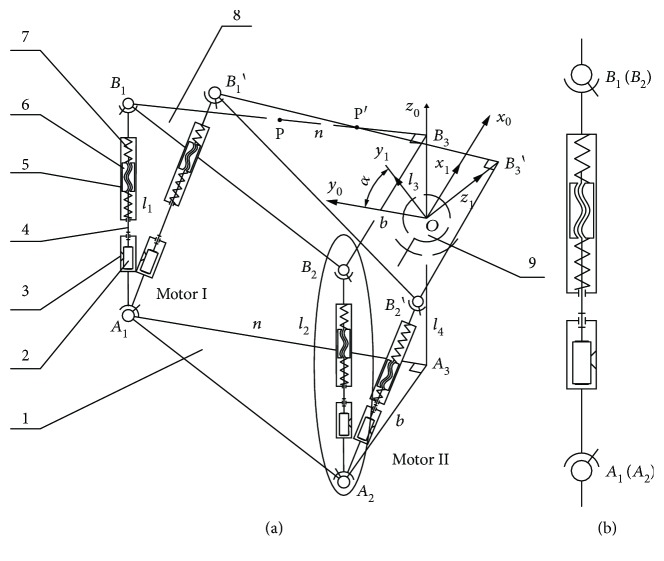
Schematic of the robot. (a) The robot rotates by an angle of *α* around the *X*_0_-axis. (b) Drive branch chain. (1) Lower platform, (2) motor, (3) U-shaped connector, (4) screw rod, (5) guide frame, (6) slider block, (7) spring, (8) upper platform, and (9) ball pin structure.

A schematic of the robot is shown in Figure 2, where (1) is the lower platform, (8) is the upper platform, and (9) is the ball pin structure supporting the two platforms. The two drive branch chains (namely, Branch Chain 1, *A*_1_*B*_1_ and Branch Chain 2, *A*_2_*B*_2_) are identical; each branched chain consists of a motor (2), U-shaped connector (3), screw rod (4), guide frame (5), slider block (6), and spring (7). The screw rod is connected to the motor, which is in turn fixed on the U-shaped connector. A screw pair is formed by the screw rod and slider block, while the slider block (6) and guide frame (5) form a sliding pair. Springs are installed between the slider block and guide frame forming the flexible transmission structure.

The guide frame (5) and the upper platform (8) and the lower platform (1) and the U-shaped connector (3) are connected by ball pin pairs, respectively. Points *A*_1_, *B*_1_, *A*_2_, *B*_2_, and *O* are centre points of the ball pin pairs, and lines *B*_1_*B*_3_ and *A*_1_*A*_3_ are perpendicular to lines *B*_3_*B*_2_ and *A*_3_*A*_2_, respectively. It is required that the spherical pin shafts of the ball pin pair *A*_1_ and *B*_1_ lie on the *A*_1_*B*_1_*B*_3_*A*_3_ plane. The spherical pin shafts of ball pin pair *A*_2_ and *B*_2_ lie on the *A*_2_*B*_2_*B*_3_*A*_3_ plane, and the spherical pin shaft of ball pin *O* lies on the *A*_1_*B*_1_*B*_3_*A*_3_ plane.

**Figure 3:**
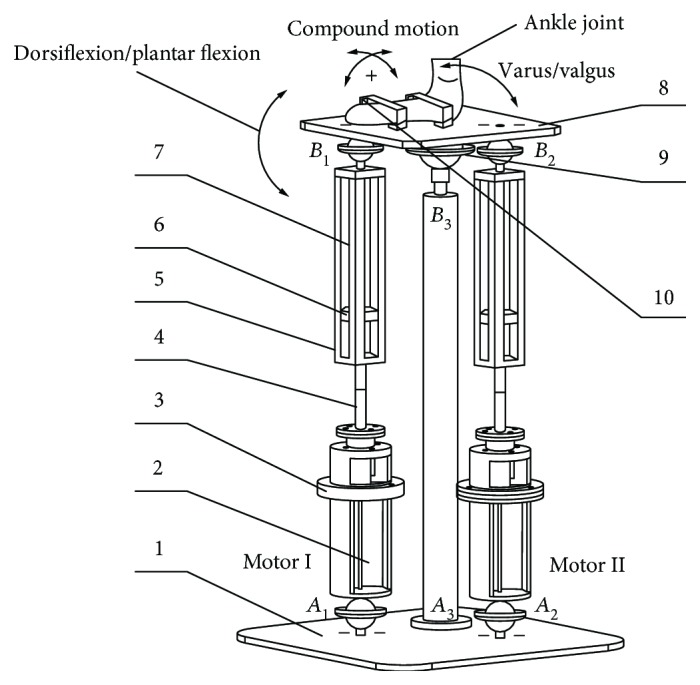
The structural model of the robot: (1) lower platform, (2) motor, (3) U-shaped connector, (4) screw rod, (5) guide frame, (6) slider block, (7) spring, (8) upper platform, (9) ball pin structure, and (10) foot buckle.

The structural model of the robot is shown in Figure 3. The foot joint of the patient is buckled on the upper platform (8) using the foot buckle (10). A structure consisting of the upper platform and the centric ball pin pair is a mirror image of a patient's foot and ankle joint. When an ankle joint needs to perform the dorsiflexion and plantar flexion rehabilitation motions, Motor I starts rotating the screw rod (4) and drives the slider block (6). Then, the motion of the slider block (6) constricts the spring (7). Under the action of the spring force, the upper platform rotates by a certain angle along the direction of the dorsiflexion and plantar flexion rehabilitation motions.

For the dorsiflexion/plantar flexion motion, only Motor I on branch chain *A*_1_*B*_1_ is needed to drive the robot (when Motor I rotates to change branch chain *A*_1_*B*_1_ for making the dorsiflexion/plantar flexion motion, the branch chain *A*_2_*B*_2_ will change its length to fit the upper platform motion. The compression of springs on branch chain *A*_2_*B*_2_ is enough to compensate for this kind of change). For the varus/valgus rehabilitation motion, the robot operates in the same way but only Motor II on branch chain *A*_2_*B*_2_ drives the robot. For the compound rehabilitation motion, both motors drive the device.

Suppose the patient uses the robot to carry out a rehabilitation motion, the movement time of the dorsiflexion/plantar flexion, varus/valgus rehabilitation, and compound rehabilitation is the same, while the motor power expense for the dorsiflexion/plantar flexion and varus/valgus rehabilitation is the same. Compared with the undecoupled robot motion system, in normal conditions, the robot can reduce energy consumption of the motor by 30%.

## 3. Motion Modelling

The following three cases can be considered when analyzing the overall motion of the system:

**Case 1 .**

Only Motor I rotates.

**Case 2 .**

Only Motor II rotates.

**Case 3 .**

Motor I and Motor II rotate simultaneously.

Each of the above cases can be divided into three modes:

1. A transition mode (i.e., the motor rotates and compresses the spring but cannot drive the motion of the upper platform)
2. A rigid-flexible combination driving mode (i.e., the motor rotates and compresses the spring, which drives the upper platform)
3. A rigid driving mode (i.e., the motor rotates and the spring is compressed to a rigid body, which drives the upper platform)

The transition mode is not considered, and the rigid driving mode cannot occur in a normal working state. Case 1 is used as an example to analyze the motion of the robot.

From Figure 2, *B*_1_, *B*_2_, and *B*_3_ are the initial positions of the upper platform, while *B*_1_′, *B*_2_′, and *B*_3_′ are the corresponding final positions.

In the initial state, let |*A*_1_*B*_1_| = *l*_1_, |*A*_2_*B*_2_| = *l*_2_, |*OB*_3_| = *l*_3_, |*A*_1_*A*_3_| = |*B*_1_*B*_3_| = *n*, and |*A*_2_*A*_3_| = |*B*_2_*B*_3_| = *b*. Set a fixed coordinate system *X*_0_*Y*_0_*Z*_0_ with the centre point *O* of the centre ball pin as the origin. The coordinate system is fixed to the lower platform. *X*_0_-axis is parallel to *A*_2_*A*_3_, *Y*_0_-axis is parallel to *A*_1_*A*_3_, and *Z*_0_-axis coincides with *OA*_3_. The coordinates of the points on the lower platform are ^0^*A*_1_(0, *n*, −*l*_4_), ^0^*A*_2_(*b*, 0, −*l*_4_), and ^0^*A*_3_(0, 0, −*l*_4_). A motion coordinate system *X*_1_*Y*_1_*Z*_1_ is set up with point *O* as the origin, and the coordinate system is fixed to the upper platform. The *X*_1_-axis is parallel to *B*_2_*B*_3_, the *Y*_1_-axis is parallel to *B*_1_*B*_3_, and the *Z*_1_-axis coincides with *OA*_3_. The coordinates of the points on the upper platform are ^1^*B*_1_(0, *n*, *l*_3_), ^1^*B*_2_(*b*, 0, *l*_3_), and ^1^*B*_3_(0, 0, *l*_3_). Due to the symmetrical structure of the upper platform, it is considered approximately that the mass centre point of the upper platform is point *P* in the middle of line *B*_1_*B*_3_ and its coordinates are ^1^*P*(0, *n*/2, *l*_3_). In the initial state, the two coordinate systems *X*_0_*Y*_0_*Z*_0_ and *X*_1_*Y*_1_*Z*_1_ are coincident.

According to Figure 2, when the upper platform rotates by an angle *α*° around the shaft *X*_0_, the homogeneous transformation matrix is given by

(1) T10=10000cosα−sinα00sinαcosα00001.

We have ^0^*B*′_1_ = _1_^0^*T* · ^1^*B*_1_, ^0^*B*′_2_ = _1_^0^*T* · ^1^*B*_2_, ^0^*B*′_3_ = _1_^0^*T* · ^1^*B*_3_, and ^0^*P*′ = _1_^0^*T* · ^1^*P*.

In order to calculate relationship between the motor drive angle and the motion angle of the upper platform, the calculation steps are as follows.

(1) *Calculating Initial Compression Displacements of the Four Springs*. Displacements *x*_12_ and *x*_22_ and *x*_32_ and *x*_42_ represent the initial compression displacements of the upper spring and the lower spring of Branch Chain 1 and Branch Chain 2, respectively. According to the forces and loads on the upper platform, establish equilibrium equations for forces and torques and the initial compression displacements *x*_12_, *x*_22_, *x*_32_, and *x*_42_ of the four springs can be calculated
(2) *Calculating Acceleration of the Mass Centre PointP*. When Motor I rotates for a time *t*(s), the upper platform rotates by an angle *α*° around the shaft *X*_0_ with the centre point *O*, and the rotation angle of Motor I is about *φ*_10_.
The acceleration P′¨O of the mass centre point *P* (see Figure 2) for the upper platform is as follows:
  (2) X¨ oP/=0,Y¨ oP/=−ncos α·α˙2+sin α·α¨2+l3sin α·α˙2+cos α·α¨,Z¨ oP/=n−sin α·α˙2+cos α·α¨2−l3cos α·α˙2+sin α·α¨,
  where *α*, $\dot{\alpha }$, and $\ddot{\alpha }$ are the angle displacement, angle velocity, and angle acceleration of the upper platform rotation motion, respectively
(3) *Calculating Acting Force between Branch Chain 1 and the Upper Platform*. The force between Branch Chain 1 and the upper platform is *F*_B13_, rotational inertia of the upper platform around the *X*_0_-axis is *J*_*X*_, and weight of the upper platform is *m*. Considering the inertia force and inertia moment of each part of Branch Chain 1, the equilibrium equation is established and *F*_B13_ is solved
(4) *Calculating Acting Force of the Upper Spring and the Lower Spring of Branch Chain 1*. In the *A*_1_*B*_1_′ direction, suppose the slider block rises Δ*x*_1_ and the guide frame rises Δ*x*_2_. Then, the compression value (see Figure 2) for the spring is Δ*x*_1_ − Δ*x*_2_. Therefore, we have
  (3) $$\begin{array}{c} \left\{\begin{array}{c} F_{13}=F_{13}^{\prime }=K\left[x_{12}+\left(\Delta x_{1}-\Delta x_{2}\right)\right],\\ F_{23}=F_{23}^{\prime }=K\left[x_{22}-\left(\Delta x_{1}-\Delta x_{2}\right)\right], \end{array}\right. \end{array}$$
  where *F*_13_ and *F*_13_′ are the forces of the upper spring of Branch Chain 1 on the guide frame and on the guide block, respectively; *F*_23_ and *F*_23_′ are the forces of the lower spring of Branch Chain 1 on the guide frame and on the slider block, respectively; and *k* is the elastic coefficient of the spring. The elastic coefficients for the upper spring and the lower spring are assumed to be the same.
Establish the differential equation for guide frame (5). According to the initial conditions, *t* = 0, Δ*x*_1_ = 0, and Δ*x*_2_ = 0, we have
  (4) $$\begin{array}{c} \Delta x_{2}=-\frac{E_{2}\cos\left(\sqrt{2K/m_{1}}t\right)}{2K}+\frac{E_{2}}{2K}, \end{array}$$
  where *E*_2_ = 2*K* · Δ*x*_1_ + *K*(*x*_12_ − *x*_22_) − *F*_B13_′ − *A*_12_ · *G*_1_. *F*_B13_′ represents the reaction force of the upper platform on the guide frame, *F*_B13_′ = −*F*_B13_; *m*_1_ is the mass of the slider block; *G*_1_ is the gravity of the slider block; $A_{12}=n\sin\alpha +l_{3}\cos\alpha +l_{4}/\sqrt{{\left(n\cos\alpha -l_{3}\sin\alpha -n\right)}^{2}+{\left(n\sin\alpha +l_{3}\cos\alpha +l_{4}\right)}^{2}}$; *n*, *l*_3_, and *l*_4_ are the structural parameters of the robot; and *α* is the angle displacement of the upper platform rotation motion
(5) *Calculating Angle Relation between the Upper Platform Motion and the Motor Drive*. When Motor I rotates by an angle *φ*_10_ for a time *t*(s), and the moving displacement of the slider block is Δ*x*_1_ = *φ*_10_*s*_*n*_/2*π*, then *s*_*n*_ is the screw pitch of the screw rod (4).
Thus, we can obtain
  (5) $$\begin{array}{c} \varphi_{10}=\frac{D_{12}-l_{1}-\left[\left(x_{12}-x_{22}\right)/2-F_{\text{B}13}^{\prime }/2K-G_{1}\cdot A_{12}/2K\right]\left(1-\cos\sqrt{2K/m_{1}}t\right)}{s_{n}\left(1-\cos\sqrt{2K/m_{1}}t\right)/2\pi }, \end{array}$$
  where *t* ≠ 0, $D_{12}=\sqrt{{\left(n\cos\alpha -l_{3}\sin\alpha -n\right)}^{2}+{\left(n\sin\alpha +l_{3}\cos\alpha +l_{4}\right)}^{2}}$, and *F*_B13_′ = −*F*_B13_. Other parameters are the same as those for equations (2), (3), and (4)

## 4. Solving the Workspace

We use the movement locus of the centre point *P* on the upper platform to express workspaces of the upper platform. For solving the workspace, the numerical method and analytical method are combined. Taking Motor I as an example, with the upper platform rotating by an angle *α*°, calculate the lengths *l*_1_(*α*) and *l*_2_(*α*) of Branch Chains 1 and 2, respectively, at a given angle and evaluate whether or not *l*_1_(*α*) and *l*_2_(*α*) are between the shortest and longest ranges of allowed branch chains. If they are within an attainable range, Branch Chains 1 and 2 with lengths *l*_1_(*α*) and *l*_2_(*α*), respectively, may form a position of the upper platform. By continuously changing the angle *α*° and evaluating the results, diverse positions for the upper platform can be obtained, corresponding to the workspace of the upper platform when Motor I runs. Similarly, the workspace for Motor II can be obtained. For solving the workspace when Motors I and II work jointly, first, the working space for each motor running solely must be obtained. Then, the two working spaces are aggregated.

### 4.1. Structure Constraints of the Branch Chain

**Figure 4:**
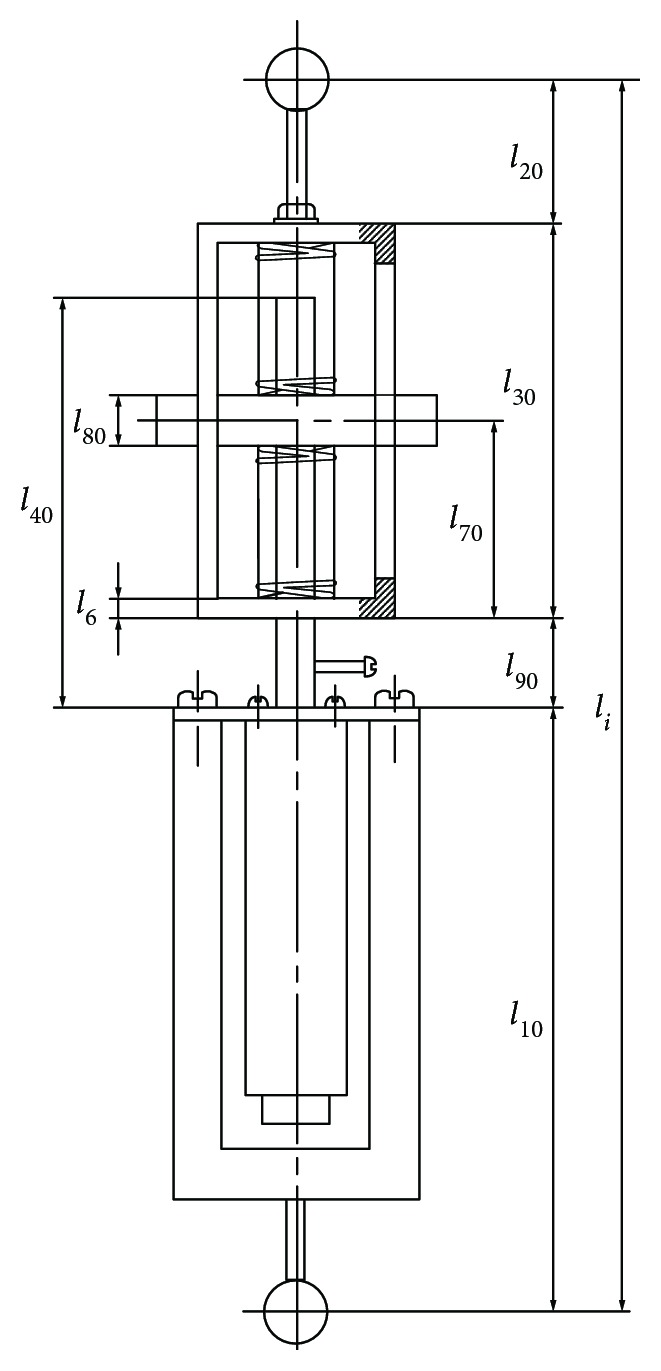
Structural model of the individual branch chain.

The structural model of a branch chain is shown in Figure 4.

Its overall length is *l*_*i*_ (hereafter referred to as the rod length), solid length of the upper spring is *l*_50_, solid length of the lower spring is *l*_60_, length of the guide frame is *l*_30_, and distance between the top of the guide frame and the upper platform is *l*_20_. Distance between the U-shaped connector and the lower platform is *l*_10_, length of the screw rod is *l*_40_, and width of the slider block is *l*_80_. At the initial position, the length between the centre of the slider block and the lower edge of the guide frame is *l*_70_, distance between the lower edge of the guide frame and the upper edge of the U-shaped connector is *l*_90_, and *l*_6_ is the thickness of the guide frame.

### 4.2. Limit Angles of the Upper Platform for Solving the Workspace

In our research, a workspace computational model is established using the workspace of the centre point *P* on the upper platform as the robot's workspace; the dorsiflexion/plantar flexion motion is taken as an example to explain limit angles of the upper platform for solving the workspace.

(1) When Branch Chain I determines the motion, the maximum and minimum angles meet the following conditions:
  (6) $$\begin{array}{c} {\left(l_{10}+l_{40}+l_{20}+l_{6}\right)}^{2}={\left(n\cos\alpha_{\min}-l_{3}\sin\alpha_{\min}-n\right)}^{2}+{\left(n\sin\alpha_{\min}+l_{3}\cos\alpha_{\min}+l_{4}\right)}^{2}, \end{array}$$
  (7) $$\begin{array}{c} {\left(l_{10}+l_{40}-\varepsilon_{l40}-l_{80}-l_{50}+l_{30}+l_{20}\right)}^{2}={\left(n\cos\alpha_{\max}-l_{3}\sin\alpha_{\max}-n\right)}^{2}+{\left(n\sin\alpha_{\max}+l_{3}\cos\alpha_{\max}+l_{4}\right)}^{2} \end{array}$$
(2) When Branch Chain II determines the motion, the maximum and minimum angles meet the following conditions:
  (8) $$\begin{array}{c} {\left(l_{10}+l_{40}+l_{20}+l_{6}\right)}^{2}={\left(l_{3}\sin\alpha_{\min}\right)}^{2}+{\left(l_{3}\cos\alpha_{\min}+l_{4}\right)}^{2}, \end{array}$$
  (9) $$\begin{array}{c} {\left(l_{10}+l_{90}+l_{70}-\frac{l_{80}}{2}-l_{50}-l_{6}+l_{30}+l_{20}\right)}^{2}={\left(l_{3}\sin\alpha_{\max}\right)}^{2}+{\left(l_{3}\cos\alpha_{\max}+l_{4}\right)}^{2} \end{array}$$

The minimum rotation angles are calculated by equations (6) and (8), and the maximum rotation angles are calculated by equations (7) and (9). For the calculation results, the absolute value of the minimum or the maximum angle is a limit angle of the upper platform for the dorsiflexion/plantar flexion motion.

### 4.3. Calculation Examples and Discussion

Based on the above analysis, a solving system for the workspace is established. Let *n* = 150, *b* = 150, *l*_10_ = 282, *l*_20_ = 33, *l*_30_ = 295, *l*_40_ = 289, *ε*_l40_ = 2, *l*_50_ = 63, *l*_60_ = 24, *l*_6_ = 10, *l*_70_ = 73, *l*_80_ = 15, and *l*_90_ = 94. In the dorsiflexion/plantar flexion motion, *α*_max_ = 30° and *α*_min_ = −30°, and in the varus/valgus motion, *β*_max_ = 30° and *β*_min_ = −30°. We study the changes of ankle posture in rehabilitation motion.

**Figure 5:**
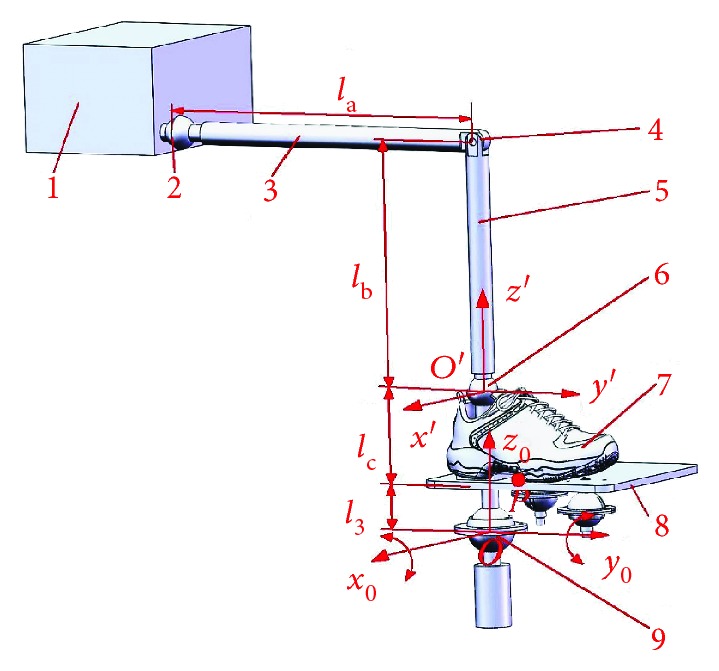
A posture model of ankle joint rehabilitation motion: (1) body, (2) hip joint, (3) thigh, (4) knee joint, (5) shank, (6) ankle joint, (7) foot, (8) upper platform, and (9) ball pin structure.

Take adult males in China as an example: according to the National Report on Nutrition and Chronic Diseases of Chinese Residents and New National Standard of Human Dimensions of Chinese Adults, the adult male has a thigh length of 465 mm, a shank length of 369 mm, and a medial malleolus height of 112 mm [29, 30]. Based on the human dimensions of Chinese adults, establish a posture model of ankle joint rehabilitation motion as shown in Figure 5 and make simulation analysis in SOLIDWORKS.

In Figure 5, the dimensions are a thigh length of *l*_*a*_ = 465 mm, a shank length of *l*_*b*_ = 369 mm, a medial malleolus height of *l*_*c*_ = 127 mm, and ball pin structure height *l*_3_ = 45 mm.

**Figure 6:**
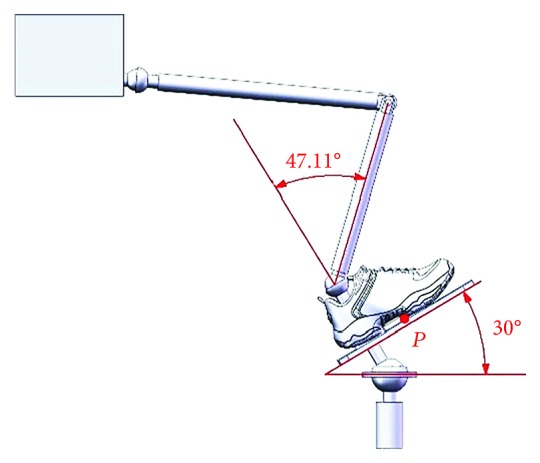
Limit posture of the ankle joint for the dorsiflexion motion (*α*_max_ = 30°).

**Figure 7:**
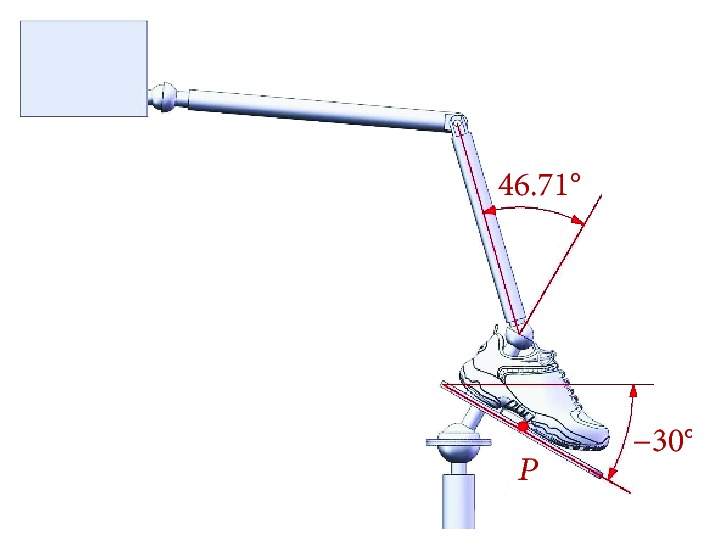
Limit posture of the ankle joint for the plantar flexion motion (*α*_max_ = −30°).

When an ankle joint carries out the dorsiflexion/plantar flexion motion, the upper platform is driven by Motor I and rotates around the *X*-axis at *α*_max_ = 30° and *α*_min_ = −30°; limit postures of the ankle joint for the dorsiflexion motion and the plantar flexion motion are as shown in Figures 6 and 7.

**Figure 8:**
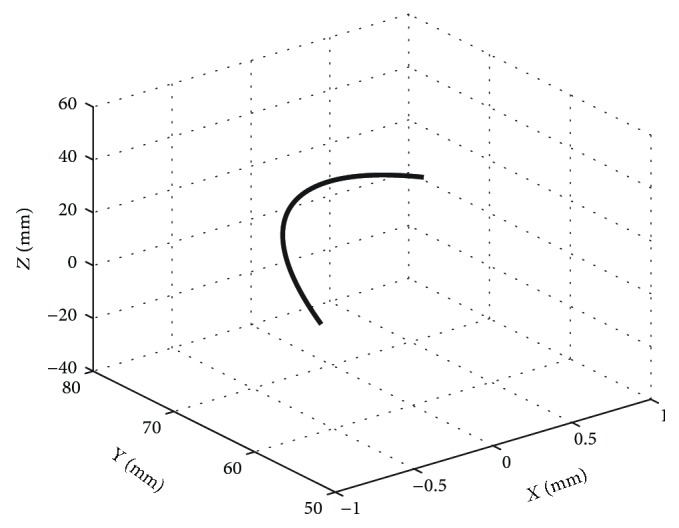
Working space for the dorsiflexion/plantar flexion motion.

Here, we use the movement locus of mass centre point *P* on the upper platform to express the workspaces of the upper platform. The workspaces are shown in Figure 8 for the dorsiflexion/plantar flexion motion.

**Figure 9:**
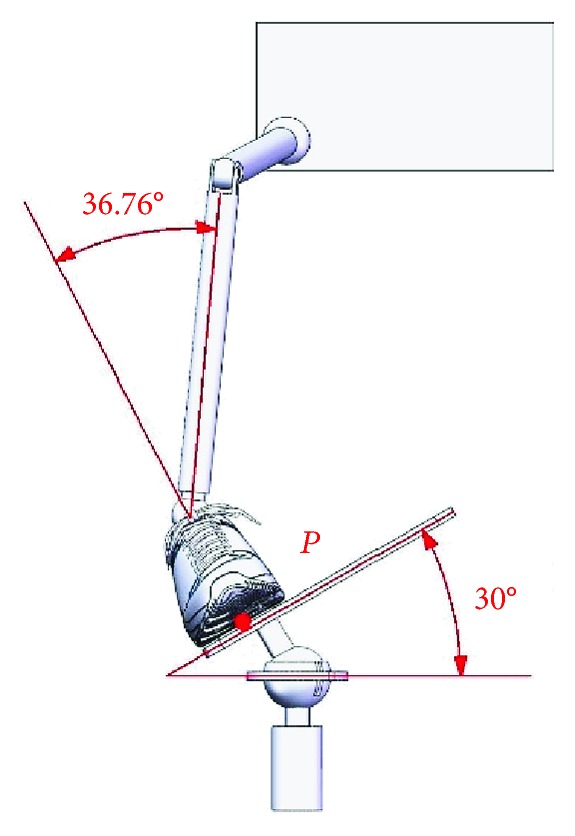
Limit posture of the ankle joint for the varus motion (*β*_max_ = 30°).

**Figure 10:**
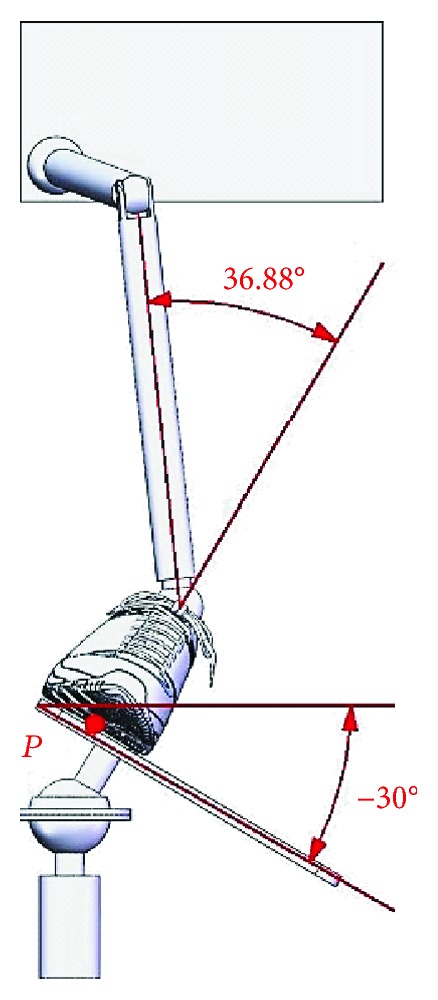
Limit posture of the ankle joint for the valgus motion (*β*_min_ = −30°).

**Figure 11:**
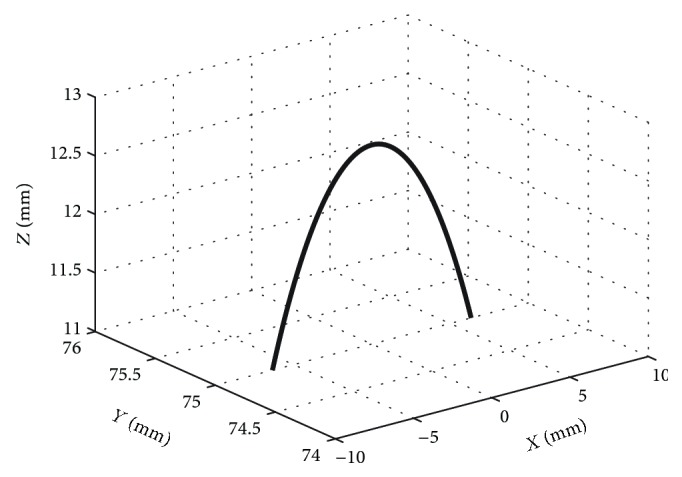
Working space for the varus/valgus motion.

When an ankle joint carries out the varus/valgus motion, the upper platform is driven by Motor II and rotates around the *Y*-axis at *β*_max_ = 30° and *β*_min_ = −30°; limit postures of the ankle joint for the varus motion and valgus motion are as shown in Figures 9 and 10. The workspaces are shown in Figure 11 for the varus/valgus motion.

**Figure 12:**
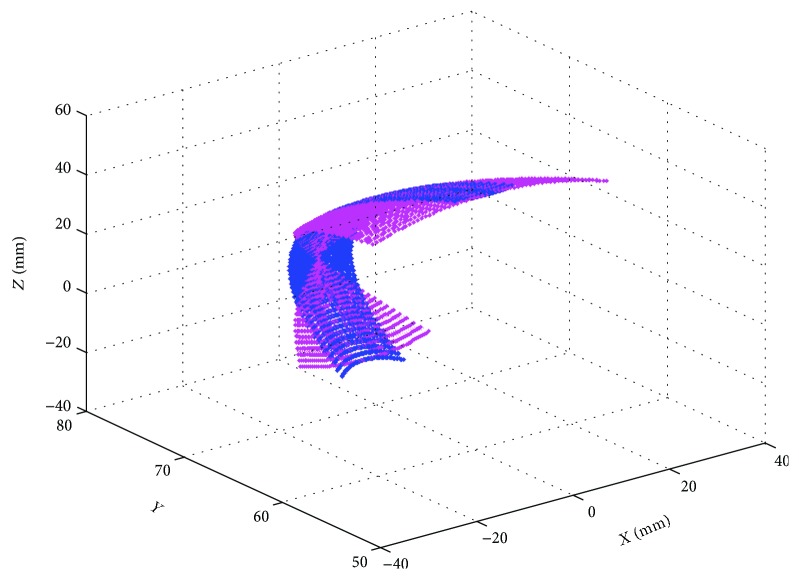
Working space for Motor I and Motor II working simultaneously.

When an ankle joint carries out the compound motion, the robot is driven by the associated motion of Motor I and Motor II. The workspaces of the upper platform are shown in Figure 12.

## 5. Motion Simulation for the Upper Platform Driven by Different Motion Laws

The performance of the robot is studied using the following three motion laws of the upper platform: modified trapezoid, modified constant velocity, and modified sine motion law [31].

Motion parameters are treated by dimensionless processing. The terms *t*, *s*, *v*, and *a* are the time, displacement, velocity, and acceleration, respectively, of the motion laws. The terms *T*, *S*, *V*, and *A* are the corresponding dimensionless parameters, and the following relationship can be established:

(10) $$\begin{array}{c} T=\frac{t}{t_{h}},\\ S=\frac{s}{h},\\ V=\frac{dS}{dT}=\frac{t_{h}}{h}v,\\ A=\frac{d^{2}S}{{dT}^{2}}=\frac{t_{h}^{2}}{h}a, \end{array}$$

where *h* and *t*_*h*_ are the total displacement and total time of the motion phase, respectively; time *t* varies in [0, *t*_*h*_], and when *t* = *t*_*h*_, *s* = *h*. Ranges of *T* and *S* are [0, 1].

**Figure 13:**
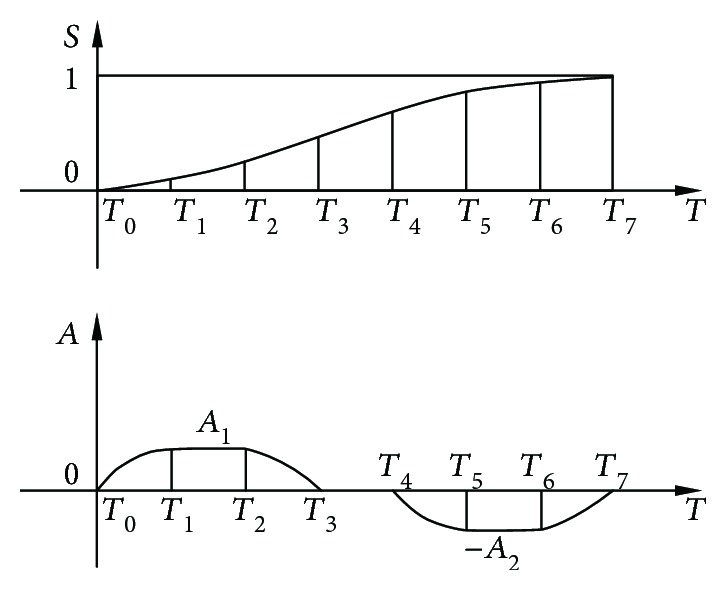
A general harmonic trapezoidal curve.

Figure 13 shows a general harmonic trapezoidal motion law expressed in dimensionless quantities.

The curve is composed of seven sections, and the acceleration of each segment is expressed as

(11) $$\begin{array}{c} A=\left\{\begin{array}{cc} A_{1}\sin\left(\frac{T}{T_{1}}\cdot \frac{\pi }{2}\right) & \left(0\leq T\leq T_{1}\right),\\ A_{1} & \left(T_{1}<T\leq T_{2}\right),\\ A_{1}\cos\left(\frac{\pi \left(T-T_{2}\right)}{2\left(T_{3}-T_{2}\right)}\right) & \left(T_{2}<T\leq T_{3}\right),\\ 0 & \left(T_{3}<T\leq T_{4}\right),\\ -A_{2}\sin\left(\frac{\pi \left(T-T_{4}\right)}{2\left(T_{5}-T_{4}\right)}\right) & \left(T_{4}<T\leq T_{5}\right),\\ -A_{2} & \left(T_{5}<T\leq T_{6}\right),\\ -A_{2}\cos\left(\frac{\pi \left(T-T_{6}\right)}{2\left(T_{7}-T_{6}\right)}\right) & \left(T_{6}<T\leq T_{7}\right), \end{array}\right. \end{array}$$

(1) By choosing different *T*_*i*_, the three motion laws listed in Table 2 can be obtained. For *T* = *T*_*i*_, according to equation (11), we have
  (12) $$\begin{array}{c} A=A_{i}=A\left(T_{i}\right). \end{array}$$
By integrating equation (12) twice, and substituting the boundary condition, i.e., *T* = 0, *S* = 0, and *V* = 0 and *T* = 1, *S* = 1, and *V* = 0, and the continuous variation conditions of the motion variables in motion process, we have
  (13) $$\begin{array}{c} S=S_{i}=S\left(T_{i}\right) \end{array}$$
(2) In order to calculate a motor drive function, the maximum motion angle of the upper platform is *α*_max_ and time is *t*_*h*_. At time *t*_*i*_(0 ≤ *t*_*i*_ ≤ *t*_*h*_), according to *T*_*i*_ = *t*_*i*_/*t*_*h*_, we have *T*_*i*_

According to the motion law chosen for the upper platform, *S*_*i*_ is calculated by equation (13) and the motion angle *α*_*i*_ of the upper platform is calculated as follows:

(14) $$\begin{array}{c} \alpha_{i}=S_{i}\alpha_{\max}. \end{array}$$

Then, equation (14) is substituted into equation (5), and a relationship between the rotating angle *φ*_10_ of the motor and the time *t* can be calculated.

Elastic coefficients of the upper spring and the lower spring might differ in the driven branch chain. To simplify the problem, when calculating the driving function of the motor, the elastic coefficients of the upper spring and the lower spring are selected with an identical value.

ADAMS software was used to simulate the motion of the upper platform. The parameters are as follows: *n* = 150 mm, *b* = 150 mm, *l*_1_ = 704.1 mm, *l*_3_ = 12.7 mm, and *l*_4_ = 691.4 mm; load on the upper platform is 2 kg; rotational inertia circling around *X*-axis is *J*_*X*_ = 43.175 kg·mm^2^; elasticity coefficient of the upper springs is *K* = 5.5125 N/mm; elasticity coefficient of the lower springs is *K* = 7.4059 N/mm; screw pitch of the screw rod is *s*_*n*_ = 5 mm; and weights of the upper platform and guide frame are *m* = 6.537 kg and *m*_1_ = 1.269 kg, respectively. Here, only the simulation analysis of the dorsiflexion/plantar flexion motion is given.

**Figure 14:**
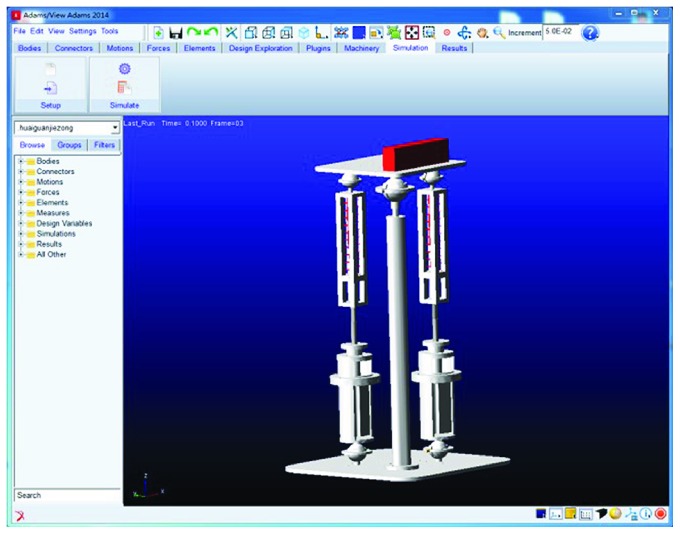
Importing the model into ADAMS.

The three-dimensional model of the robot is imported into ADAMS software (Figure 14). Revolving joint motion around the *Z*-axis is added to the motor to simulate the motor's rotation.

When only Motor І rotates, the upper platform is loading and the simulation is given here. In the work process, the upper platform adopts the modified trapezoid, the modified constant velocity, and the modified sinusoidal motion laws.

A cuboid whose outline size is 220 × 60 × 40 mm (*L* × *H* × *W*) is added to the upper platform, and a 2 kg mass is set to simulate the patient's foot. The simulation time of the upward motion (i.e., *α* changes from 0° to 30°) or downward motion (*α* changes from 0° to -30°) of the upper platform is 5 s in steps of 0.1 s.

**Figure 15:**
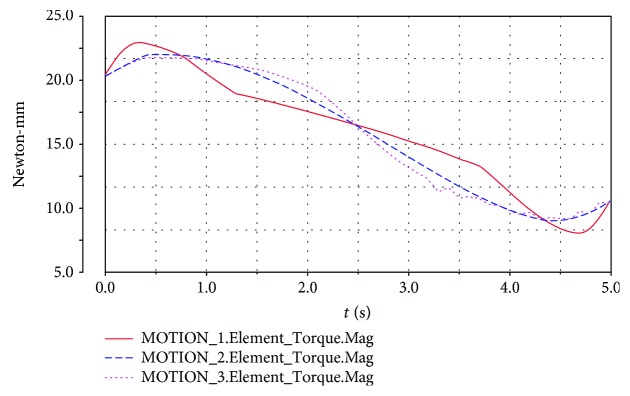
Torque for the upper platform moving upward. MOTION_1: modified constant velocity function; MOTION_2: modified sine function, MOTION_3: modified trapezoidal function.

**Figure 16:**
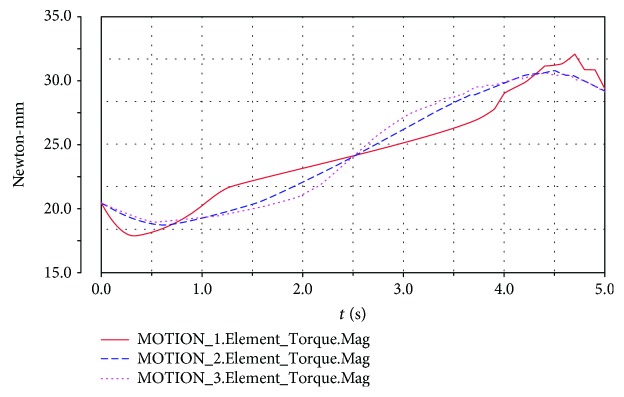
Torque for the upper platform moving downward. MOTION_5: modified constant velocity function; MOTION_6: modified sine function; MOTION_7: modified trapezoidal function.

The upper platform is driven by motion laws previously established, and the motor torque changes are shown in Figures 15 and 16 when the upper platform moves from the equilibrium position upward to the top position and downward to the lowest position, respectively.

When the motion is driven by the modified trapezoidal function or the modified constant velocity function, the torque values of the motor fluctuate at the beginning, middle, and end of the motion. The use of the modified sine function enjoys better results than the other two kinds of driving function.

When the spring is set to the elastic state and the rigid state, simulation analysis is carried out. Some of the simulated parameters are summarized in Table 3.

For the spring in the elastic state, the maximum angular velocity is 0.234 rad/s and the maximum angular acceleration is -1.290 rad/s^2^, whereas for the spring in the rigid state, the maximum angular velocity is 0.229 rad/s and the maximum angular acceleration is -0.593 rad/s^2^. The maximum angular velocity value of the upper platform moving with the same motion law is larger for the spring in the case of the elastic state than that for the spring in the case of the rigid state. Moreover, the maximum angular acceleration value of the upper platform is significantly higher for the spring in the case of the elastic state. However, the maximum value of the torque does not significantly differ for the elastic state and the rigid state. Therefore, in the rehabilitation exercise, the patient can choose the different laws of motion based on the specific rehabilitation needs.

## 6. Control System and Prototype Experiment

### 6.1. Control System Scheme

**Figure 17:**
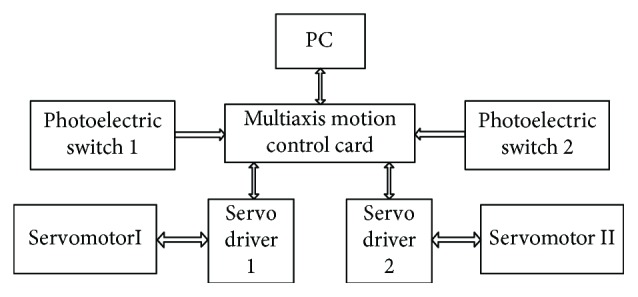
Schematic of the overall control system.

The control system shown in Figure 17 is composed of a PC, a multiaxis motion control card, and servo drive control systems. The PC provides the user with a graphical interface to complete different tasks such as the motion parameter setting. The multiaxis motion control card obtains the instructions and then converts them into the corresponding signals. The servo driver receives the corresponding signals and drives the servomotor.

### 6.2. Prototype Test

**Figure 18:**
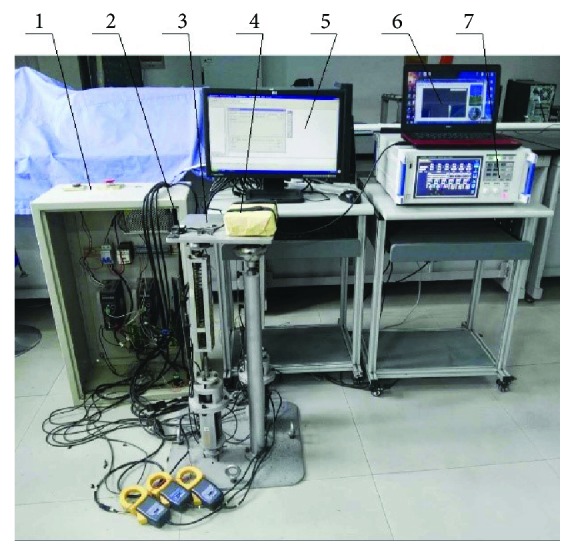
Experimental prototype of the robot and measurement system: (1) control cabinet, (2) gyro, (3) prototype of the robot, (4) load simulating patient foot, (5) PC for the control system of the robot, (6) PC for the measurement system, and (7) power analyzer.

We design a pose measurement system. The measurement system uses a gyro accelerometer MPU6050 to measure the motion angle of the upper platform and a power analyzer HIOKI PW6001 to measure the currents and power of the motor. Measurement data is shown through the PC. The measurement system can display the upper platform movement in three-dimensional angle changes.  Table 4 summarizes the main technical parameters of the servomotor used. The robot experimental prototype and the measurement system are shown in Figure 18.

To compare experiment results with the simulation results using ADAMS software, a cuboid load with overall dimensions of 190 × 130 × 50 mm (*L* × *H* × *W*) and weight of 2 kg is added on the platform to simulate the patient foot.

#### 6.2.1. Single Motor Drives

**Figure 19:**
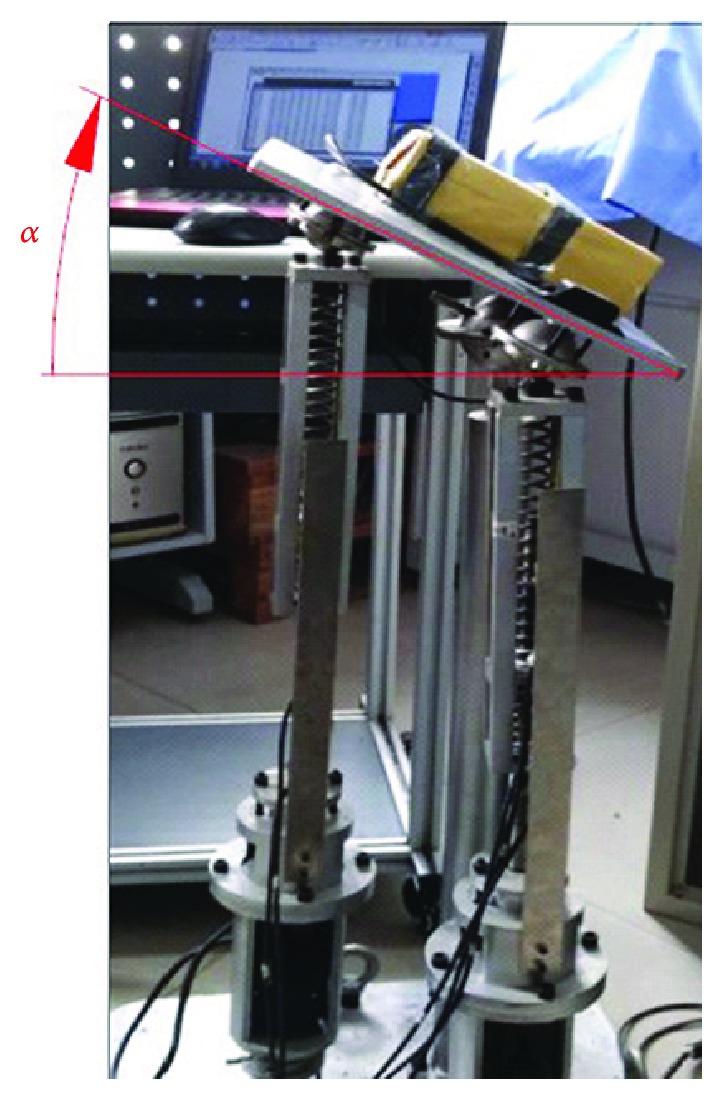
The experiment for a single motor drive.

Figure 19 shows the angle changes of the upper platform when the robot is driven by Motor I (see Figure 3) to realize the dorsiflexion/plantar flexion movement.  Table 5 shows the angle changes of the upper platform.

From Figure 19 and Table 5, the upper platform only conducts angle changes needed for the dorsiflexion/plantar flexion movement. Experiments for the varus/valgus movement have the same result. Those experiments show that the experimental prototype of the robot can realize drive motion decoupling.

#### 6.2.2. The Compound Motion

**Figure 20:**
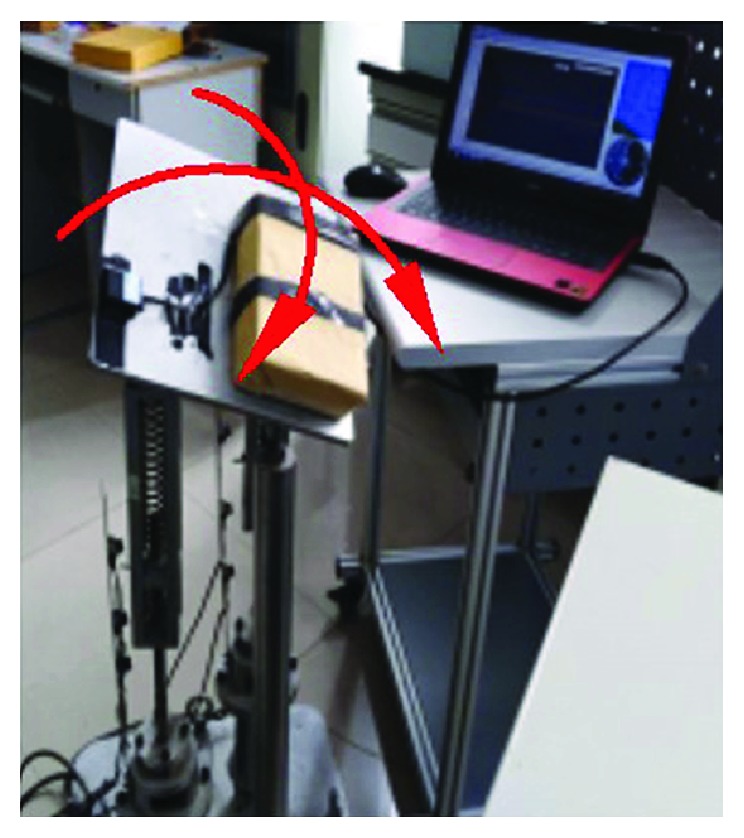
The experiment for the compound motion.

Realizing the compound motion is tested by using two motor drives.  Figure 20 shows the angle changes of the upper platform when the robot is driven using two motors. From Figure 20, the upper platform can conduct the angle changes needed for the compound motion.

#### 6.2.3. The Maximum Working Angles for the Upper Platform

According to the design parameters, the maximum working angles for the dorsiflexion/plantar flexion motion or the varus/valgus motion change from -30° to +30°. Actual maximum working angles for the upper platform are tested. The experiment shows that the maximum working angles meet the design requirements.

**Figure 21:**
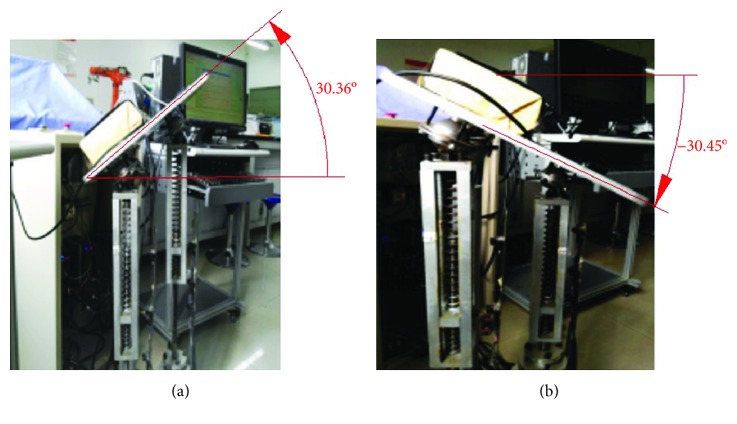
The maximum working angles for the varus/valgus rehabilitation motion: (a) move up and (b) move down.

The maximum working angles for the varus/valgus rehabilitation motion according to the modified sine motion law are shown in Figure 21.

#### 6.2.4. The Real-Time Process of Rehabilitation Motion

**Figure 22:**
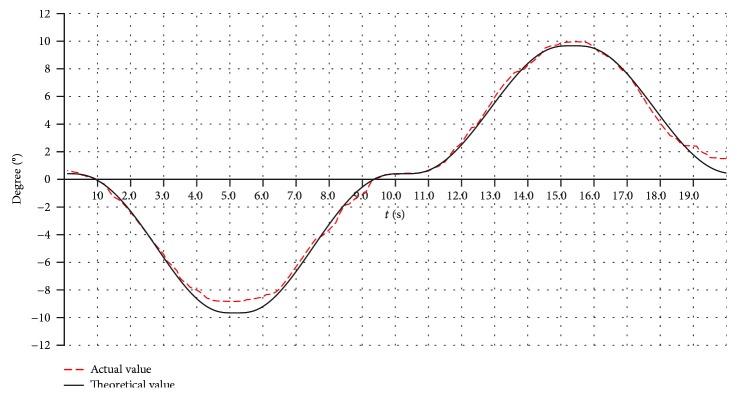
The working angles (from -10° to +10°) for the varus/valgus rehabilitation motion.

**Figure 23:**
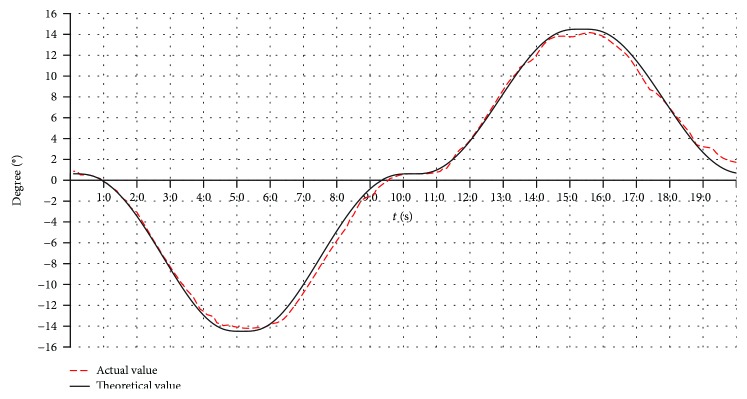
The working angles (from -15° to +15°) for the varus/valgus rehabilitation motion.

**Figure 24:**
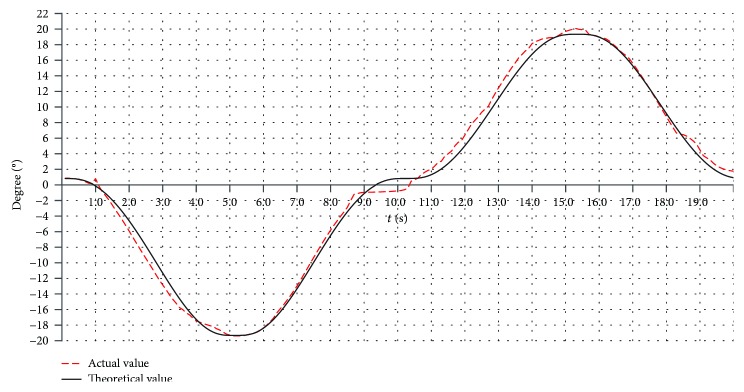
The working angles (from -20° to +20°) for the varus/valgus rehabilitation motion.

The varus/valgus motion, the dorsiflexion/plantar flexion motion, and the compound motion are tested. Here, only the varus/valgus motions are used as an example. The rehabilitation motion of the upper platform is driven by the modified sine motion law, and the cycle time is 20 s. The experiment results for three working angles (changing from -10° to +10°, -15° to +15°, and -20° to +20°) are shown in Figures 22, 23, and 24.

The theoretical values in Figures 22, 23, and 24 are simulated using ADAMS software. From the test results, we found that the overall trends of the actual value were consistent with the simulation results.

The actual working angles deviate from the ideal value between -1.7° and +1.6°, when the working angles change from -10° to +10° as shown in Figure 22. The actual working angles deviate from the ideal value by -1.2° to+1.0°, when the working angles change from -15° to +15° as shown in Figure 23. The actual working angles deviate from the ideal value by -1.1° to+0.6°, when the working angles change from -20° to +20° as shown in Figure 24.

**Figure 25:**
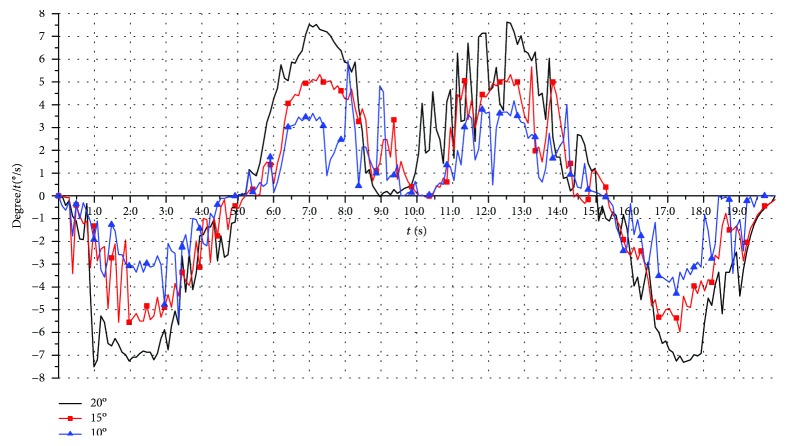
The working velocities for the varus/valgus rehabilitation motion.

The experiment result for the three working velocities corresponding to the angles changing in Figures 22, 23, and 24 is shown in Figure 25. The characteristic value of the working velocities is shown in Table 6. The actual velocity values are obtained by differential calculation from the actual working angle change values. While from Figure 25 the varus/valgus rehabilitation motion is not smooth, there are some velocity fluctuations. From Table 6, theoretical values of the velocity are obtained by calculating from the modified sine motion law used by the upper platform motion; the actual testing maximum value and minimum value of the velocity are larger than the ones of the theoretical velocity. The test results show that the speed fluctuates greatly when the upper platform moves to the extreme position and horizontal position. This result is caused by the rigid-flexible hybrid structure of the robot. The spring is subjected to the pressing force which causes it to fluctuate in the abovementioned stage, causing deformation fluctuations.

**Figure 26:**
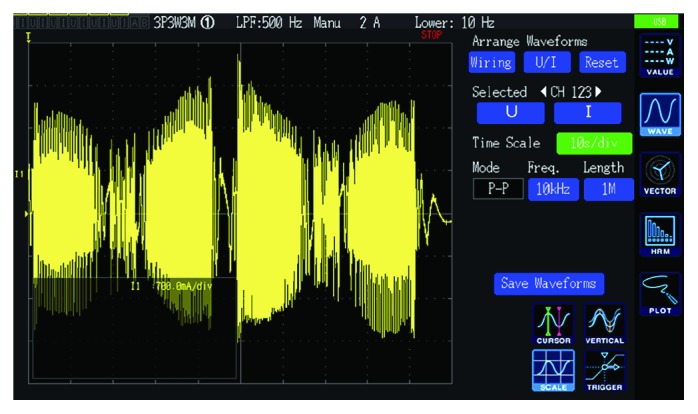
A working interface of the power analyzer.

**Figure 27:**
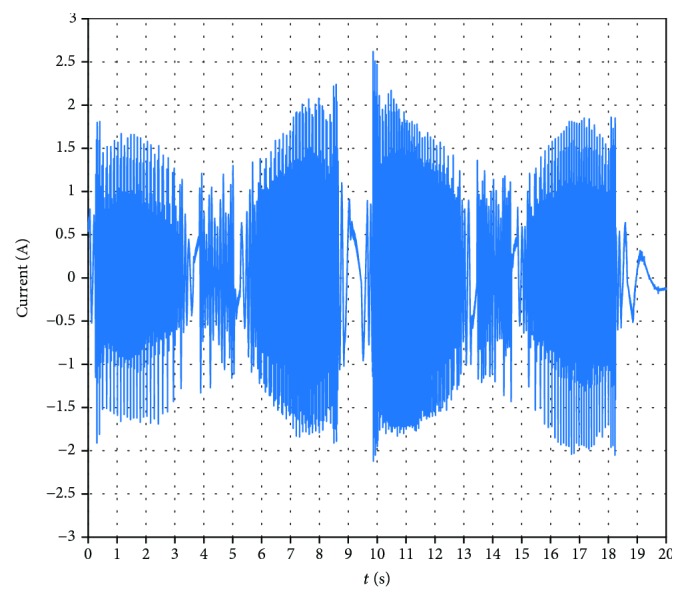
The working currents of the motor for the varus/valgus rehabilitation motion (from -20° to +20°).

**Figure 28:**
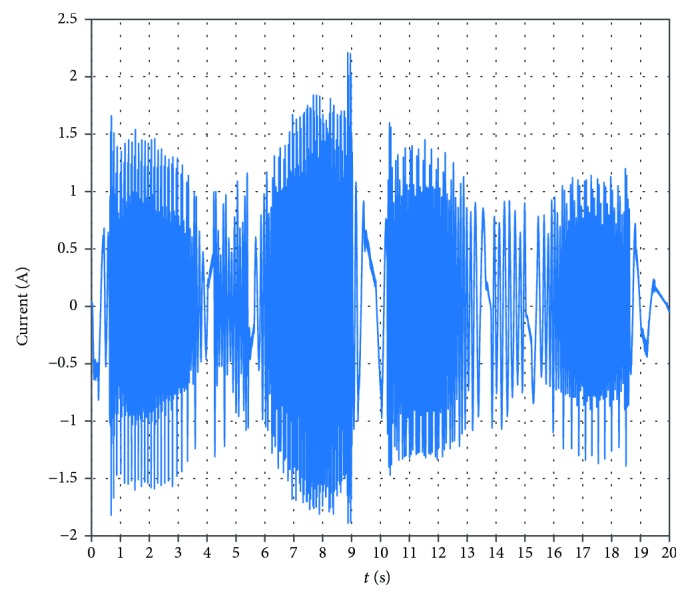
The working currents of the motor for the Varus/valgus rehabilitation motion (from -10° to +10°).

A power analyzer Hioki PW6001 is used to measure the working currents of the motor. A working interface of the power analyzer is shown in Figure 26. The working currents of the motor for the varus/valgus rehabilitation motion (from -20° to +20°) are shown in Figure 27. The maximum value of the currents is 2.62 A. The working currents of the motor for the varus/valgus rehabilitation motion (from -10° to +10°) are shown in Figure 28, and the maximum value of the currents is 2.21 A. From Table 4, the rated line current of the servomotor is 2.8 A, which indicates that the motor works in the normal range. The current changes periodically, and its period is basically the same with the speed period. The test results show that the current is relatively stable at 8.6-10 s. This is due to the fact that the upper platform moves close to the horizontal position and the upper platform moves at a lower speed. The load of the platform is mainly carried by the ball pin pair, and the load component of the varus/valgus branch chain is small and the change is not obvious.

According to the on-the-spot observation and test, the error between the actual values and the theoretical values may be caused by manufacturing and assembling precision for the structure, especially the manufacturing precision of the spring, the screw, etc. The performance of the spring is a critical factor.

#### 6.2.5. Rehabilitation Motion on a Human Ankle Joint

**Figure 29:**
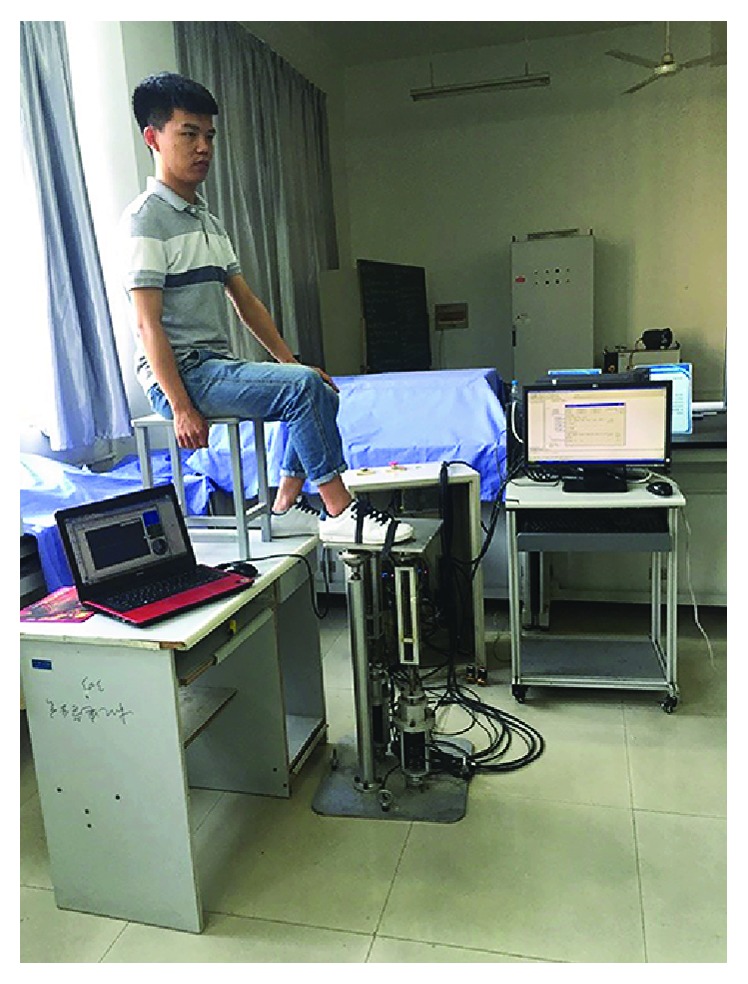
Testing the robot on a human ankle joint.

We tested the robot on a human ankle joint in the lab; the test scenario is as shown in Figure 29.

We have tested the varus/valgus motion, the dorsiflexion/plantar flexion motion, and the compound motion, separately. The rehabilitation motion of the upper platform is driven by the modified sine motion law, and the cycle time is 20 s.

**Figure 30:**
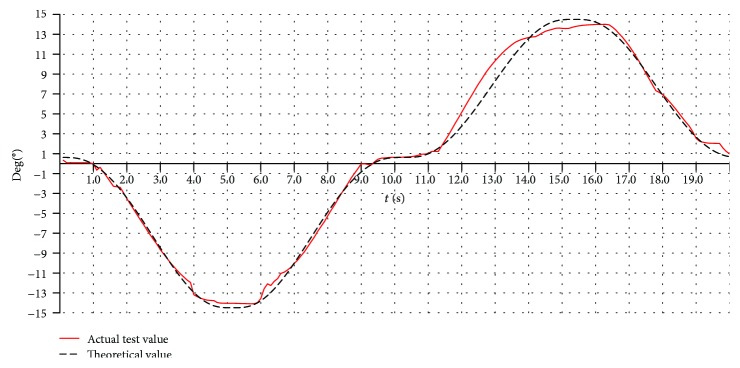
The working angles (from -15° to +15°) for the varus/valgus rehabilitation motion tested on a human ankle joint.

Here, only the varus/valgus motion (working angles changing from -15° to +15°) is used as an example. The experiment results are shown in Figure 30. The actual value deviates from the ideal value by -2.1° to +0.9°.

Analyzing the result in Figure 30, we found that the overall trends of the actual results tested on the human ankle joint are consistent with the theoretical values. Load on the upper platform for the human ankle joint is 7.2 kg. Comparing this result with the result tested on adopting the cuboid load (cuboid load is 2 kg, as shown in Figure 18), there are small differences.

A further in-depth study about clinical data is our future work target.

## 7. Conclusion

This paper presents an ankle joint rehabilitation robot with a rigid-flexible hybrid driving structure based on a 2-S′PS′ mechanism. The robot has two DOFs but can realize the three kinds of motion for the ankle joint rehabilitation.

The robot uses a centre ball pin pair as the main support to reduce the load of the drive system. The structure of the robot consisting of an upper platform and a centre ball pin pair is a mirror image of a patient's foot and ankle joint, which accords with physiological characteristics of the human body. In the dorsiflexion/plantar flexion or varus/valgus driving system, the robot adopts the rigid-flexible hybrid structure and the robot motion is completely decoupled.

The presented robot has low manufacturing and usage costs. The theoretical analysis and experimental prototype show that the robot can meet some rehabilitation needs of different patients.

## Figures and Tables

**Table 1:** Actuation, RoM and motion decoupled characteristic of ankle rehabilitation robots.

Year	Authors	DOF	RoM	Actuator	Motion decouples
2006	Liu et al. [22]	3	41.9° plantarflexion	Electric motor	No
			43.8° dorsiflexion		
			42.8° abduction		
			41.9° abduction		
			53.8° inversion		
			44.1° eversion		

2006	Yoon et al. [23]	2	50° plantarflexion/dorsiflexion	Pneumatic actuator	No
			55° inversion/eversion		

2009	Saglia et al. [24]	2	30° dorsiflexion	Electric motor	No
			60° plantarflexion		
			30° inversion		
			15° eversion		

2009-2014	Jamwal et al. [25, 26]	3	46° plantarflexion/dorsiflexion	Pneumatic actuator	No
			52° abduction/adduction		
			26° inversion/eversion		

2010	Ding et al. [27]	2	45° plantarflexion/dorsiflexion	Magneto-rheological fluid (MRF)	No
			12° inversion/eversion		

2013	Bi [9]	3	99.50° inversion/eversion	Electric motor	No
			56.00° dorsiflexion/plantarflexion		
			100.80° internal/external rotations		

2018	Liao et al. [13]	3	75° plantar/dorsal flexion	Electric motor	No
			42° inversion/eversion		

2018	C.D. Wang (author of this paper)	2	60° dorsiflexion/plantar flexion	Electric motor	Yes
			60° varus/valgus		

**Table 2:** Different motion laws.

	*T*_0_	*T*_1_	*T*_2_	*T*_3_	*T*_4_	*T*_5_	*T*_6_	*T*_7_
Modified trapezoid motion law	0	1/8	3/8	1/2	1/2	5/8	7/8	1
Modified sinusoidal motion law	0	1/8	1/8	1/2	1/2	7/8	7/8	1
Modified constant velocity motion law	0	1/16	1/16	1/4	3/4	15/16	15/16	1

**Table 3:** Analysis results of maximum angular speed and acceleration of the platform and maximum motor torque.

Curve		Project					
		Maximum angular velocity (rad/s)		Maximum angular acceleration (rad/s^2^)		Motor maximum torque (N·mm)	
		Upward motion	Downward motion	Upward motion	Downward motion	Upward motion	Downward motion
Spring in the elastic state	Modified trapezoid	0.234	-0.223	-0.756	0.503	0.382	0.534
	Modified constant velocity	0.164	-0.155	-0.222	-1.290	0.401	0.560
	Modified sinusoidal	0.208	-0.197	0.200	0.440	0.384	0.538

Spring in the rigid state	Modified trapezoid	0.229	-0.222	0.414	-0.538	0.387	0.539
	Modified constant velocity	0.157	-0.150	-0.209	-0.555	0.407	0.559
	Modified sinusoidal	0.201	-0.194	-0.593	-0.197	0.391	0.541

**Table 4:** Technical parameters of the servomotor.

Category	Parameter	Category	Parameter
Motor model	ACH-06040DC	Maximum torque	3.8 N·m
Rated power	400 W	Rated line current	2.8 A
Rated speed	3000 r/min	Rated line voltage	220 V
Rated torque	1.27 N·m	Number of encoder lines	2500 PPR

**Table 5:** Angle changes for the upper platform.

Number	*α* (°)	*β* (°)	*γ* (°)
	Around the shaft *X*_0_	Around the shaft *Y*_0_	Around the shaft *Z*_0_
1	-2.5763	0.0165	-0.0055
2	-3.3618	0.0275	-0.0055
3	-4.1473	0.0385	-0.0055
4	-5.0098	0.0439	-0.0055
5	-5.8667	0.0494	-0.0110
6	-6.8005	0.0439	-0.0055
7	-7.7069	0.0439	-0.0055
8	-8.5034	0.0604	-0.0110
9	-9.2889	0.0769	-0.0110
10	-9.9207	0.0989	-0.0165
11	-10.5414	0.1099	-0.0165
12	-11.0083	0.1263	-0.0220
13	-11.4203	0.1373	-0.0220

**Table 6:** The angular velocity characteristic value changes for the upper platform.

Working angles (°)	-20° to +20°		-15° to +15°		-10° to +10°	
Angular velocity (°/s)	Theoretical value	Actual value	Theoretical value	Actual value	Theoretical value	Actual value
Maximum value	6.978	7.624	5.233	5.658	3.489	4.175
Minimum value	-6.978	-7.503	-5.233	-5.966	-3.489	-3.850

## Data Availability

The data used to support the findings of this study are available from the corresponding author upon request.

## Nomenclature

P′¨O: Acceleration of the mass centre point *P*
*x*_12_: The initial compression displacements of the upper spring of Branch Chain 1
*x*_22_: The initial compression displacements of the lower spring of Branch Chain 1
*x*_32_: The initial compression displacements of the upper spring of Branch Chain 2
*x*_42_: The initial compression displacements of the lower spring of Branch Chain 2
*F*_B13_: The force between Branch Chain 1 and the upper platform
*F*_B13_′: The reaction force of the upper platform on the guide frame
*J*_*X*_: The rotational inertia of the upper platform around the *X*_0_-axis
*m*: The weight of the upper platform
Δ*x*_1_: The rising displacement of the slider block
Δ*x*_2_: The rising displacement of the guide frame
*F*_13_: The force of the upper spring of Branch Chain 1 on the guide frame
*F*_13_′: The force of the upper spring of Branch Chain 1 on the guide block
*F*_23_: The force of the lower spring of Branch Chain 1 on the guide frame
*F*_23_′: The forces of the lower spring of Branch Chain 1 on the slider block
*K*: The elastic coefficient of the spring
*m*_1_: The mass of the slider block
*G*_1_: The gravity of the slider block
*n*: The structural size of the upper platform
*b*: The structural size of the upper platform
*l*_3_: The structure height of the ball pin
*l*_4_: The length between the centre point *O* of the centre ball pin and the lower platform
*s*_*n*_: The screw pitch of the screw rod
*l*_*i*_: The overall length of the branch chain
*l*_1_: The overall length of the branch chain in the initial state
*l*_50_: The solid length of the upper spring
*l*_60_: The solid length of the lower spring
*l*_30_: The length of the guide frame
*l*_20_: The distance between the top of the guide frame and the upper platform
*l*_10_: The distance between the U-shaped connector and the lower platform
*l*_40_: The length of the screw rod
*l*_80_: The width of the slider block
*l*_70_: The length between the centre of the slider block and the lower edge of the guide frame at the initial position
*l*_90_: The distance between the lower edge of the guide frame and the upper edge of the U-shaped connector
*l*_6_: The thickness of the guide frame
*l*_*a*_: The length of a thigh
*l*_*b*_: The length of a shank
*l*_*c*_: The height of the medial malleolus
*h*: The total displacement of the motion phase
*t*_*h*_: The total time of the motion phase.
